# Low-dose shift- and rotation-invariant diffraction recognition imaging

**DOI:** 10.1038/s41598-022-15486-y

**Published:** 2022-07-01

**Authors:** Tatiana Latychevskaia, Alice Kohli

**Affiliations:** 1 Paul Scherrer Institute, Forschungsstrasse 111, 5232 Villigen, Switzerland; 2 Physics Department, University of Zurich, Winterthurerstrasse 190, 8057 Zurich, Switzerland

**Keywords:** Imaging and sensing, Microscopy, Imaging techniques

## Abstract

A low-dose imaging technique which uses recognition rather than recording of a full high-resolution image is proposed. A structural hypothesis is verified by probing the object with only a few particles (photons, electrons). Each scattered particle is detected in the far field and its position on the detector is analysed by applying Bayesian statistics. Already a few detected particles are sufficient to confirm a structural hypothesis at a probability exceeding 95%. As an example, the method is demonstrated as an application in optical character recognition, where a hand-written number is recognized from a set of different written numbers. In other provided examples, the structural hypothesis of a single macromolecule is recognized from a diffraction pattern acquired at an extremely low radiation dose, less than one X-ray photon or electron per Å^2^, thus leaving the macromolecule practically without any radiation damage. The proposed principle of low-dose recognition can be utilized in various applications, ranging from optical character recognition and optical security elements to recognizing a certain protein or its conformation.

## Introduction

One can recognize a familiar object or scene from only a few details without a need for an entire high-resolution image. Thus, a few measurements can be sufficient to extract all the relevant information. The task of recognition finds applications in different areas. In optics, for example, optical character recognition (OCR) allows for identifying the correct symbol from a set of available symbols. Although OCR can be routinely performed by commercially available devices, it is still a challenge to identify a character from a noisy and low-quality image, one solution here is to apply neural networks^1^. In biology, molecular structures can be simulated based on theoretical models in great detail. The purpose of an experiment can thus be replaced from obtaining a full high-resolution image of macromolecule to recognizing a certain molecular structure. The recent breakthrough in solving the protein folding problem using artificial intelligence (Alphafold) has already provided a large number of models to the three-dimensional (3D) shapes of proteins^2^. At the same time, the experimental imaging of a single protein still remains a challenge due to the radiation damage problem^3^. Most protein structures are being solved using X-ray crystallography and cryo-electron microscopy, and are the result of averaging over tens of thousands of molecules. Presently, there is a strong wish to develop experimental techniques which would allow atomic resolution imaging of truly individual molecules^4–14^. However, radiation damage is the main limiting factor^15^, since a macromolecule is destroyed long before a sufficient number of scattering events is detected for a high-resolution structural analysis^3,4^. Alternative methods for structural determination which minimize the radiation dose have recently been proposed, they allow for the verification of a structural hypothesis with just a few scattering events. In these schemes, a quantum sorter is designed based on the molecular structure hypothesis and is positioned between the macromolecule and the detector^16,17^. Although highly promising, the experimental realization of such methods has been troubled due to several factors, mainly due to the practical limits in nano-fabrication of the structure-defined diffractive elements and the necessity of an atomic-precision alignment of the sorter caused by the high sensitivity of the method to lateral shifts and rotations of either the molecule or the sorter (the latter is illustrated in Fig. S1).

Here, we propose a low-dose diffraction recognition imaging which identifies the correct object from a set of hypotheses by using Bayesian statistics analysis. Two-dimensional (2D) diffraction pattern is replaced with one-dimensional (1D) probability function obtained from azimuthal-averaged diffraction pattern, which solves the two issues in the quantum recognition schemes^16,17^: no sorting element is needed and object can be recognized in any in-plane orientation. The method is independent of the lateral position and in-plane rotation of the object. We demonstrate an application of the method for optical character recognition (OCR)^18^, where a hand-written character is recognized from a set of different written characters. We also provide an example of recognizing the structural hypothesis of a single macromolecule's orientation from its diffraction pattern. It is shown that already a few detected particles are sufficient to confirm a structural hypothesis at a high probability exceeding 95%.

## Principle

### Low-dose recognition imaging

For *M* object hypotheses, *M* corresponding images $I_{i} \left( p \right)$ are given and the corresponding probability density functions (PDFs) are calculated as:

1 $$P\left( {p\left| I \right._{i} } \right) = \frac{{I_{i} \left( p \right)}}{{\sum\limits_{p = 1}^{N} {I_{i} \left( p \right)} }},$$

where *p* is the pixel coordinate and *N* is the total number of pixels. For a 2D diffraction pattern, *p* is running through the $\left( {q_{x} ,q_{y} } \right)$ coordinates in the far-field detector plane $\left( {q_{x} ,q_{y} } \right)$. The a priori probabilities of having any of the given hypotheses are equal:

2 $$P_{1} = P_{2} = ... = P_{M} = \frac{1}{M}.$$

After the first particle is detected in pixel *p*_1_*,* the a posteriori probabilities that the corresponding PDF corresponds to the imaged structure are given by

3 $$P_{i}^{^{\prime}} = \frac{{P_{i} \cdot P\left( {p_{1} \left| I \right._{i} } \right)}}{{\sum\limits_{j = 1}^{M} {\left[ {P_{j} \cdot P\left( {p_{1} \left| I \right._{j} } \right)} \right]} }}.$$

The updated probabilities become the input a priori probabilities for the analysis of the next particle detected in pixel *p*_2_. The probabilities are then updated according to Eq. (3), and so forth. Equation (3) gives $P_{1}^{^{\prime}} = P_{1} = 1$ for *M* = 1.

**Figure 1:**
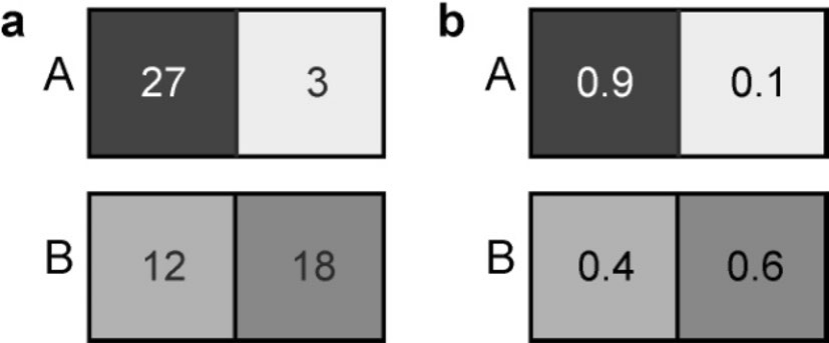
Two two-pixel images, A and B (**a**) and their corresponding probability density functions (PDFs) (**b**).

The proposed Bayesian recognition principle is illustrated using two two-pixel images, A and B with intensity distributions $I_{A} \left( p \right)$ and $I_{B} \left( p \right)$, shown in Fig. 1a.

According to Eq. (1) the PDF for each image are given by:

$$P\left( {p\left| A \right.} \right) = \frac{{I_{A} \left( p \right)}}{{\sum\limits_{p = 1}^{2} {I_{A} \left( p \right)} }}, \, P\left( {p\left| B \right.} \right) = \frac{{I_{B} \left( p \right)}}{{\sum\limits_{p = 1}^{2} {I_{B} \left( p \right)} }},$$

and they are shown in Fig. 1b. The initial probabilities that the detected image corresponds to hypothesis A or B are equal to 0.5:$P_{A} = P_{B} { = 0}{\text{.5}}$. After the first particle is detected in pixel *p*, according to Eq. (3), the probability that the observed image corresponds to hypothesis A is given by

$$P_{{\text{A}}}^{^{\prime}} = \frac{{P_{{\text{A}}} \cdot P\left( {p\left| {\text{A}} \right.} \right)}}{{P_{{\text{A}}} \cdot P\left( {p\left| {\text{A}} \right.} \right) + P_{{\text{B}}} \cdot P\left( {p\left| {\text{B}} \right.} \right)}}.$$

When the first particle is detected at pixel $p = 1$, the updated probability, according to Eq. (3) is given by:

$$P_{{\text{A}}}^{^{\prime}} = \frac{{0.5 \cdot P\left( {1\left| {\text{A}} \right.} \right)}}{{0.5 \cdot P\left( {1\left| {\text{A}} \right.} \right) + 0.5 \cdot P\left( {1\left| {\text{B}} \right.} \right)}} = \frac{0.9}{{0.9 + 0.4}} = 0.692.$$

The updated probability that the detected image corresponds to hypothesis B is given by $$P_{{\text{B}}}^{^{\prime}} = 1 - P_{{\text{A}}}^{^{\prime}} = 0.308.$$$$P_{{\text{A}}}^{^{\prime}}$$ and $$P_{{\text{B}}}^{^{\prime}}$$ are a posteriori probabilities, which are then used as input probabilities for the analysis of the next detected particle. When the second particle is again detected at pixel $$p = 1$$, the updated probability that the observed image corresponds to hypothesis A is given by:

$$P_{{\text{A}}}^{^{\prime}} = \frac{{0.692 \cdot P\left( {1\left| {\text{A}} \right.} \right)}}{{0.692 \cdot P\left( {1\left| {\text{A}} \right.} \right) + 0.308 \cdot P\left( {1\left| {\text{B}} \right.} \right)}} = \frac{0.692 \cdot 0.9}{{0.692 \cdot 0.9 + 0.308 \cdot 0.4}} = 0.835,$$

and the probability that the detected image corresponds to hypothesis B is given by $P_{{\text{B}}}^{^{\prime}} = 0.165.$ And so forth, the routine is repeated for each next detected particle. Thus, by counting individual particles arriving at the detector and analysing their position on the detector, the probabilities of the hypotheses are quantitatively evaluated.

### Low-dose shift-invariant diffraction recognition imaging

In general, an object's image is shifted together with the object. Thus, each image of a shifted object corresponds to a different hypothesis. To create an imaging method which recognizes object independently on its lateral position, a diffraction pattern of the object can be considered instead of its image. A diffraction pattern of an object distribution is obtained by acquiring the squared amplitude of the Fourier transform (FT) of the object distribution. Shifting the object distribution creates an additional phase factor of its complex-valued Fourier transform distribution without changing its amplitude. The resulting diffraction pattern is thus independent on the object shift.

In an optical diffraction experiment, the probing particles (photons, electrons) scattered off an object give rise to a diffraction pattern in the far field. The diffraction pattern, in principle, is a distribution of the probability to detect a particle at a certain point on the detector. A probing particle, after being scattered by the object, changes its state in such a way that the probability of detecting it at a certain point on the detector in the far field is given by the diffraction pattern. A single scattered particle could arrive seemingly at any position at the detector. But when the second, the third and further scattered particles also arrive at the positions that are distributed according to a diffraction pattern corresponding to a certain sample hypothesis, there is a high probability for the structural hypothesis to be correct. In this arrangement, even a few scattered probing particles are sufficient to determine the correct structural hypothesis. While conventional statistics analysis considers the outcomes of detecting scattered particles as being independent of one another, the Bayesian statistical analysis takes into account previous events and updates the probability based on previous outcomes. Quantitatively, the normalized diffraction pattern $I\left( {q_{x} ,q_{y} } \right) = I_{0} \left( {q_{x} ,q_{y} } \right)/\iint {I_{0} \left( {q_{x} ,q_{y} } \right){\text{d}}q_{x} {\text{d}}q_{y} }$ provides a probability density function (PDF) which gives the probability of detecting the scattered particle at a position $\left( {q_{x} ,q_{y} } \right)$ on the detector; $I_{0} \left( {q_{x} ,q_{y} } \right)$ is the recorded diffraction pattern.

The recognition analysis performed on 2D diffraction patterns instead of 2D images of an object does not depend on the in-plane shift of the object. However, it still depends on the in-plane rotation of the object. When an object is rotated in-plane, its 2D diffraction pattern is also rotated by the same degree. Thus, the diffraction patterns of the object and of the rotated object are different and therefore correspond to two different structural hypotheses. The diffraction patterns of the object corresponding to its different in-plane rotations can be used as a set of hypotheses and the in-plane rotation of the object can be determined from them.

### Low-dose shift- and rotation-invariant diffraction recognition imaging

To make the recognition method independent of the in-plane rotation of the object, a 1D PDF is obtained from azimuthally-averaged 2D diffraction pattern as follows. For each $q$ value ($q = \sqrt {q_{x}^{2} + q_{y}^{2} }$), and each azimuthal angle $\vartheta$, the value of diffraction pattern at $\left( {q,\vartheta } \right)$ is extracted giving $I_{0} \left( {q,\vartheta } \right)$. The azimuthal angle values are run over 2$\pi$ in steps of $\Delta \vartheta$ and the total sum of all particles detected at a given *q* is given by

4 $$I\left( q \right) = \frac{2\pi q}{N}\sum\limits_{n = 0}^{N - 1} {I_{0} \left( {q,n\Delta \vartheta } \right)} ,$$

where $N = \frac{2\pi }{{\Delta \vartheta }}$ is the number of steps. The 1D PDF function is then calculated by normalizing $I\left( q \right)$ using Eq. (1). The obtained 1D PDF function is related only to the radial positions of the detected particles. The method is also insensitive to the lateral position of the sample, since a diffraction pattern is insensitive to the lateral (in-plane) shifts of the imaged sample. An example of 1D radial profile evolution as a function of the number of detected particles in a 2D image is provided in Fig. S2. The approach of using 1D radial profile has been previously explored in applying deep-learning techniques for structural recognition in X-ray powder diffraction data^19^.

For a 3D object in a certain orientation, its 2D diffraction pattern is approximately given by the diffraction pattern of the object's 2D projection. An out-of-plane rotation of a 3D object results in a different 2D projection and, as a result, in a different 2D diffraction pattern. A different diffraction pattern, in turn, corresponds to a different hypothesis. Different hypotheses can correspond to different out-of-plane rotations of the same 3D object, or different out-of-plane rotations of different 3D objects. Thus, the recognition process can be realized for: different out-of-plane rotations of the same 3D object, or different out-of-plane rotations of different 3D objects. Once the correct out-of plane orientation of a 3D object is determined, the in-plane rotation can be found as explained at the end of previous section: by applying recognition of the entire 2D diffraction pattern against diffraction patterns corresponding to different in-plane rotations of the object.

## Results

### Character recognition

**Figure 2:**
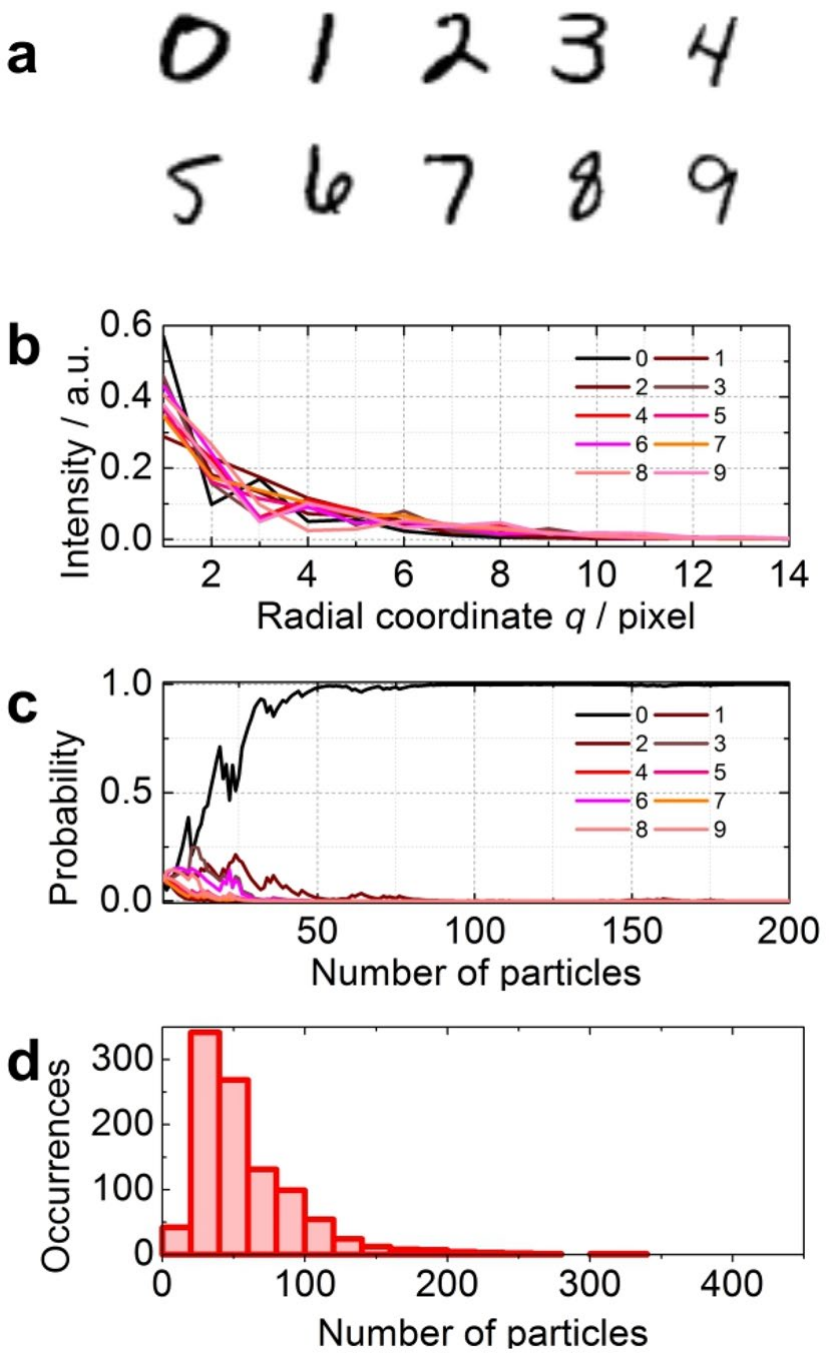
Principle of low-dose diffraction recognition imaging. (**a**) Ten samples constituting the then hypotheses. (**b**) 1D PDF profiles corresponding to the diffraction patterns of each sample. (**c**) Evolution of probabilities as a function of number of detected particles. (**d**) Number of particles required to achieve 95% probability that the sample structure hypothesis is correct; results of 1000 experiments.

The principle of the low-dose diffraction recognition imaging is demonstrated using an example of 10 images of hand-written numbers, each sampled with 28 × 28 pixels, Fig. 2a. These are the known distribution—hypotheses. For each object, its diffraction pattern was calculated, the azimuthally averaged 1D profile was extracted, and the corresponding 1D PDF was obtained, shown in Fig. 2b. In the numerical experiment, one object was selected, the corresponding diffraction pattern was calculated, and the radial distribution was obtained by azimuthal averaging of the diffraction pattern. To mimic experimental conditions, Gaussian-distributed noise was added in order to reach SNR = 2. The Gaussian-distributed noise was added as follows: at each value of signal S, an array of Gaussian-distributed noise with standard deviation S/SNR was generated using a built in routine (LabView), the noise distribution and its parameters were checked, and the first value from the array was added to the signal value. The PDF was calculated using Eq. (1). The radial positions on the detector of the particle scattered off the probed structure were modelled using Monte Carlo simulations. Each particle scattered off the sample was analysed by Bayesian analysis against the ten noise-free hypotheses using Eq. (3). According to Eq. (2), the initial probability for all hypotheses is 0.1. As the number of the detected particles increased, the probability approached 1 for the correct hypothesis, and 0 for all other hypotheses, Fig. 2c. Numerical experiments showed that based on the results of 1000 numerical experiments, about 40 particles were needed to achieve 95% confidence level of the probability that the hypothesis was correct, Fig. 2d. It was verified that this number was approximately the same for the same images sampled with different number of pixels: 28 × 28 pixels and 140 × 140 pixels. About 40 particles were needed to achieve 95% confidence level of the probability that the hypothesis was correct for each image size (Fig. S3).

The proposed low-dose recognition method allows for the recognition of a 2D object independent of the object's shift or rotation. The method can be applied, for example, in optical character recognition (OCR)^18^, where quality and noise of the images is often a problem for the successful identification of a character. The example demonstrated here shows that character recognition can be realized for noisy and arbitrarily rotated images by acquiring only 100 particles (samples) instead of acquiring full two-dimensional images.

### Conformation recognition

**Figure 3:**
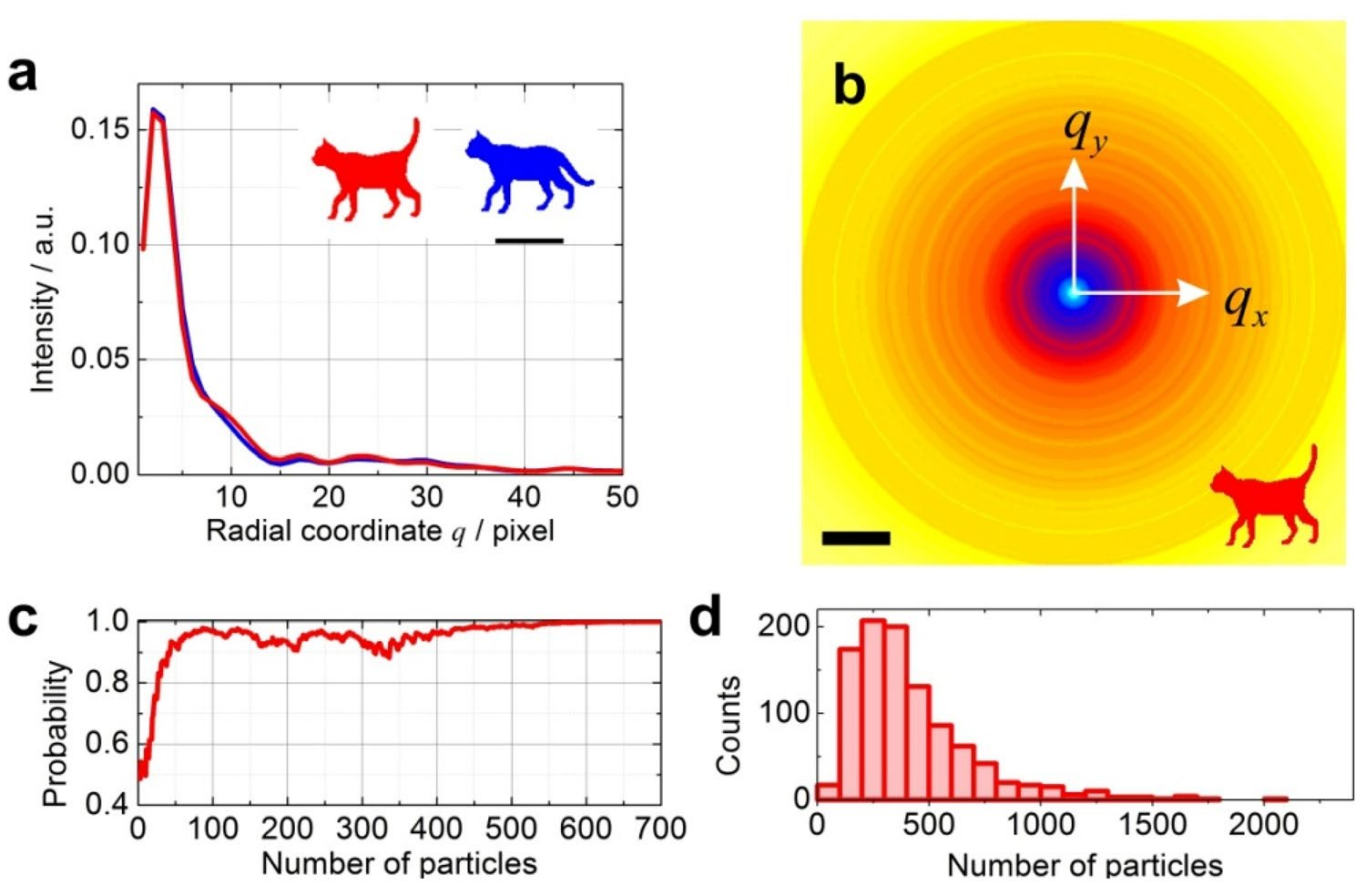
Principle of structure determination demonstrated for a binary sample – a cat cartoon. (**a**) The sample can be found in two conformations: with tail up ("up", shown in red) or tail down ("down", shown in blue). (**a**) 1D PDF profiles corresponding to the diffraction patterns; the region where two signals exhibit difference is shown. The scalebar is 60 a.u. (**b**) Azimuthally-averaged diffraction pattern of "up" conformation. The scalebar is 5 pixels in the Fourier space, with 1 pixel = 1/400 a.u. (**c**) Probability of the right hypothesis ("up") as a function of the number of detected scattered particles. (**d**) Number of particles required to achieve 95% probability that the sample structure hypothesis is correct; results of 1000 experiments are shown.

The example in this section shows how a small change in the sample can be recognized using the proposed technique. Here, a test sample is a cat cartoon in two possible conformations—with its tail up ("up") and with its tail down ("down"), Fig. 3a. 1D PDF profiles obtained from the corresponding azimuthal-averaged diffraction patterns exhibit almost identical distributions, Fig. 3a–b. The probed structure is in the "up" confirmation and it is verified against the two conformation hypotheses: "up" and "down". The 1D PDF is calculated from the radial distribution of the azimuthal-averaged diffraction patterns corresponding to the "up" conformation using Eq. (4), with noise added to reach SNR = 2. The numerical experiments show that approximately 300 particles are required to reach a 95% probability that the structural hypothesis is correct, Fig. 3c–d. Cross-correlation function (CCF) analysis is a conventional approach to quantitatively estimate whether the signal is matching a reference signal. By applying the CCF analysis to the radial distributions updated after each detected particle, we observed that both CCFs exhibit very close values with only about 10E-2 difference (Fig. S4), thus providing a less clear answer to the structural hypothesis.

### Macromolecule orientation recognition from X-ray diffraction pattern

**Figure 4:**
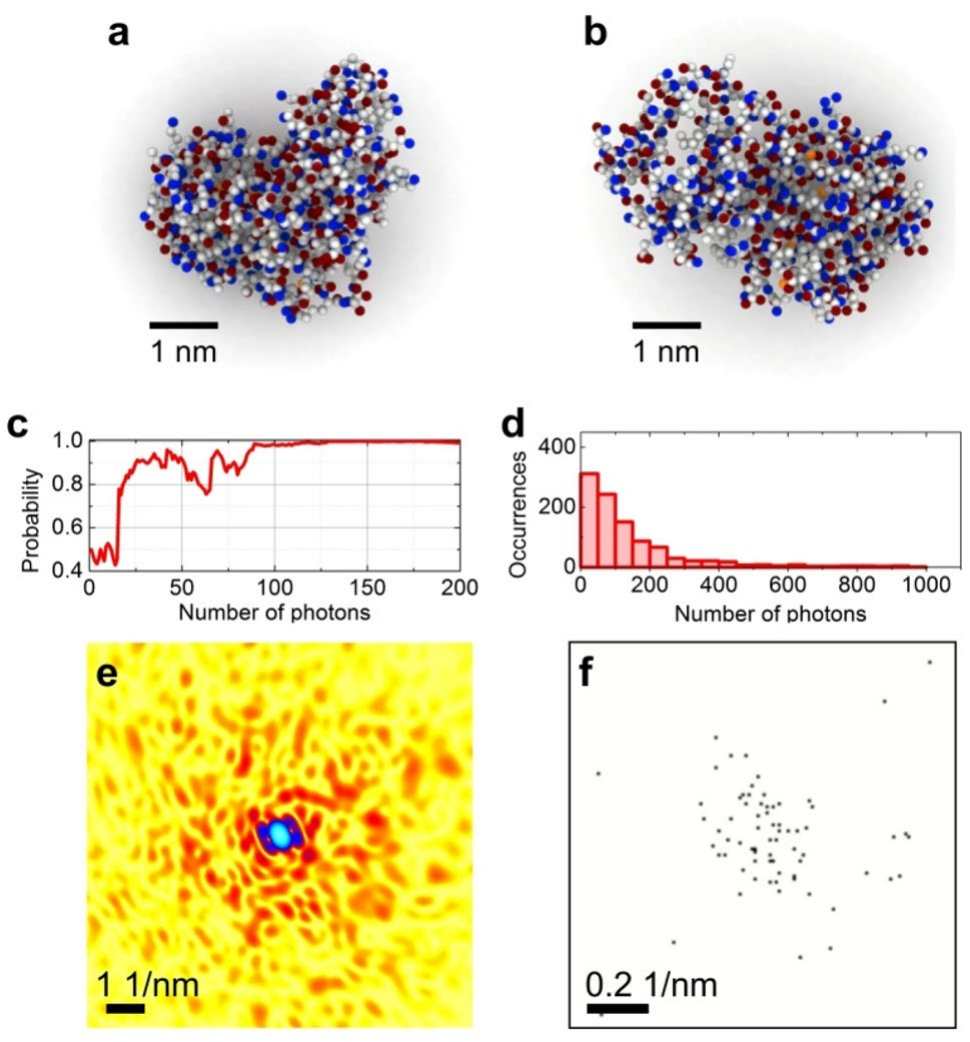
Structure verification of a single lysozyme molecule from a low-dose X-ray diffraction pattern. (**a**) and (**b**) structure of the molecule in *xy*- and *xz*-orientation, respectively. (**c**) Probability of the right hypothesis (*xy*-orientation) as a function of the number of detected photons. (**d**) Number of photons required to achieve 95% probability that the sample structure hypothesis is correct; results of 1000 experiments are shown. (e) and (f) X-ray diffraction patterns of a lysozyme molecule formed by elastically scattered (e) 1.24E + 8 photons/Å^2^ and (**f**) 0.11 photons/Å^2^ (f). In (**e**), the full, 1024 × 1024 pixel diffraction pattern is shown, the maximum is 8.0E + 7 counts per pixel (cpp). In (**f**), only the central 128 × 128 pixel region of 1024 × 1024 diffraction pattern is shown, with the maximum of 1 cpp.

The low-dose recognition method can be particularly useful for verifying the structure of radiation sensitive biological macromolecules, as for example in the quantum recognition schemes^16,17^. In the next example, a single lysozyme molecule^20,21^ is recognized from two possible hypotheses: molecule being in *xy* or *xz* orientation, Fig. 4a,b. The enzyme was set in the *xy*-orientation, Fig. 4a, and probed with X-ray photons of 1 Å wavelength. The diffraction patterns were simulated as described in Methods. The radial positions of the individual particles on the detector were simulated using the Monte Carlo technique, the 1D PDF was created in a similar way as in the previous example, with noise added to reach SNR = 2; the currently reported noise level for diffraction patterns in single particle imaging (SPI) amounts to SNR = 7^22^. Approximately 100 elastically scattered photons were needed to reach 95% probability that the structural hypothesis was correct (Fig. 4c,d). This results in 0.11 photons/Å^2^ for elastically scattered particles, or an approximate radiation dose of 1.1 photons/Å^2^, considering that only one photon out of ten is elastically scattered^3^. Thus, a structural hypothesis of a single lysozyme molecule can be verified with a radiation dose of one X-ray photon/Å^2^, which is orders of magnitudes less than typical radiation dose in high-resolution X-ray imaging^4–14^. The total number of 100 photons is extremely low. To illustrate this fact, the intensity distribution obtained with 100 photons is shown in Fig. 4e,f. This low-dose diffraction pattern exhibits almost no signal outside of the central region of 0.2 1/nm resolution, the limit which is comparable to the size of molecule itself, 5 nm. Such a low-dose electron diffraction pattern cannot be used for structure determination by an iterative phase retrieval algorithm. In the provided here example a lysozyme molecule was selected to compare the obtained results to the results presented by Neutze et al^3^ who proposed "diffract and destroy" experiment and showed that a diffraction pattern of a single lysozyme molecule would exhibit such a low number of counts per pixels at the rim (where the resolution is 2.2 Å) that iterative phase retrieval methods cannot be applied to reconstruct the molecular structure. Here we show that, alternatively, structure of an individual macromolecule can be *recognized* from its low-dose diffraction pattern.

### Macromolecule orientation recognition from electron diffraction pattern

**Figure 5:**
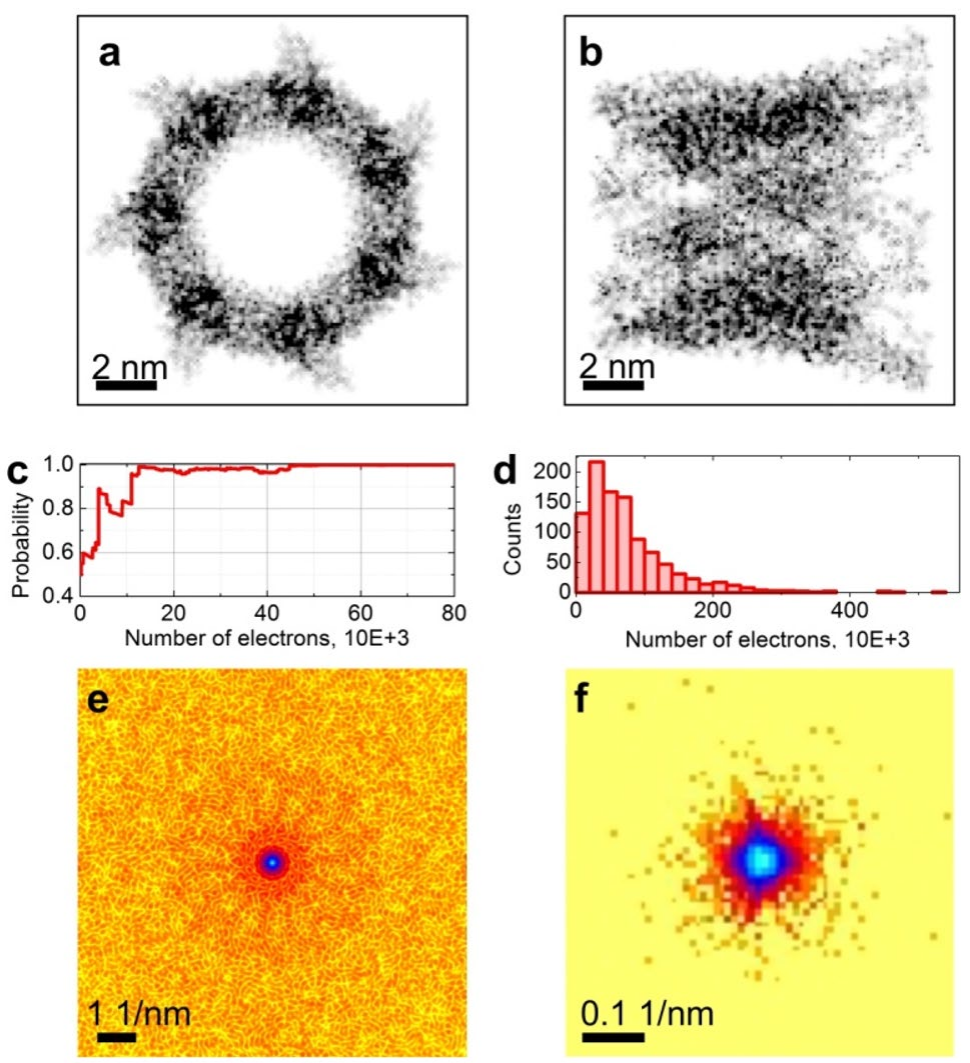
Structure verification of a single EspB protein by counting single scattered electrons. (**a**) and (**b**) the phase distributions of the exit wave are shown calculated for 200 keV electrons, the macromolecule in *xy* and *xz* orientation, respectively. The phase shift reaches 0.84 in (**a**) and 0.74 rad in (**b**). (**c**) Probability of the right hypothesis (*xy*-orientation) as a function of the number of detected electrons. (**d**) Number of electrons required to achieve 95% probability that the sample structure hypothesis is correct; results of 1000 experiments are shown. (**e**) and (**f**) diffraction patterns of a single EspB protein at a radiation dose of (**e**) 8.23E + 5 e/Å^2^ (**b**) and 48.6E-3 e/Å^2^ (**f**). (**e**) Full, 1024 × 1024 pixel diffraction pattern and (**f**) only the central 64 × 64 pixel region of 1024 × 1024 diffraction pattern are shown.

Similar results were obtained for electron diffraction of an individual macromolecule, as for example EspB, a virulence protein secreted from Mycobacterium tuberculosis^23,24^. The diffraction patterns were simulated as described in Methods. Gaussian noise was added to the diffraction pattern so that the SNR at a certain *q* value is modelled as SNR(*q*) = 1/(30*q*), where *q* is in Angstrom; for example SNR = 0.33 at *q* = 0.1 1/Å and SNR = 0.11 at q = 0.3 1/Å. This particular model of SNR(*q*) roughly approximates the experimental observations^25^. The orientation of the protein was validated against the *xy* and *xz* orientations, Fig. 5a,b. The probed molecule was positioned in the *xy*-orientation (Fig. 5a) and approximately 40′000 electrons were needed to reach 95% probability that the structural hypothesis was correct (Fig. 5c,d). For the total probed area of 8.23E + 5 Å^2^ this translates into a dose of 48.6E-3 e/Å^2^, a much smaller dose than the typical 2–5 e/Å^2^ required in high-resolution imaging cryo-electron microscopy^26–28^. The electron diffraction pattern produced with a dose of 48.6E-3 e/Å^2^ exhibits mainly a very low-resolution signal, Figs. 5e–f.

## Discussion

Low-dose diffraction recognition imaging verifies a structural hypothesis by detecting only a few scattered particles. Detecting scattered particles in the far field and analysing only their radial positions on the detector make the method invariant to shift and in-plane rotation of the object. For live low-dose imaging, the scattered particles can be detected and analysed during imaging. For experiments where only a post-experimental low-count image is available, as in the case of a diffraction pattern of a protein, each individual count in a pixel can be considered as a detected particle analysed by the proposed method as explained in the example of two-pixel images.

The proposed method can be adapted for optical image recognition, which finds applications in microscopy, medical imaging, robotic vision, optical remote sensing, and optical security methods. In optical security techniques, image recognition is currently achieved by performing correlation of a reference object and a target object to be recognized, for example by means of holography^29,30^. In the herewith provided example of OCR, a character is recognised using much less samples than when acquiring a full image of the character.

When applied for structural biology, the herewith proposed method allows for the verification of a structural hypothesis by probing the sample with only a few particles (photons, electrons). Previously, Neutze et al^3^ described their "diffract and destroy" experiment and showed that a diffraction pattern of a single biological macromolecule (lysozyme) would exhibit such a low number of counts per pixel at the rim of the diffraction pattern. Such a diffraction pattern cannot be used for high-resolution structure reconstruction by iterative phase retrieval methods. With the method proposed here we show that, alternatively, structure of an individual macromolecule can be *recognized* from a diffraction pattern, even when the diffraction pattern is acquired at a very low dose.

High-resolution imaging techniques such as cryo-EM or X-ray SPI^14,31^, conventionally employ a low-resolution model of the molecular structure as reference to perform cross-correlation analysis for alignment of the experimental images into a high-resolution image of the structure. Currently, the EMC method (E for expansion, M for maximization and C for compression), proposed by Loh and Elser, is applied for analysis of SPI data to reconstruct a particle’s three-dimensional (3D) diffraction intensity from many photon shot-noise limited two-dimensional measurements^32^.

In the method proposed here, instead of a full image only a few scattered counts are detected, thus demonstrating that the cross-correlation analysis can be replaced by the low-dose recognition method.

Another possible application of the herewith proposed method can be a sequential imaging of macromolecule undergoing conformational or dynamical changes where at each time frame the macromolecule is probed with only a few particles (photons, electrons), so that the conformational changes can be determined without significant damaging of the structure during the entire acquisition time.

We provided several examples of possible applications of the method. In general, the proposed method can be applied for any recognition task where the hypotheses exhibit different PDF distributions. This could include: different orientations of 3D objects, different 2D or 3D objects, different conformations of the same objects, etc.

## Methods

### X-ray diffraction pattern of a single lysozyme molecule

**Figure 6:**
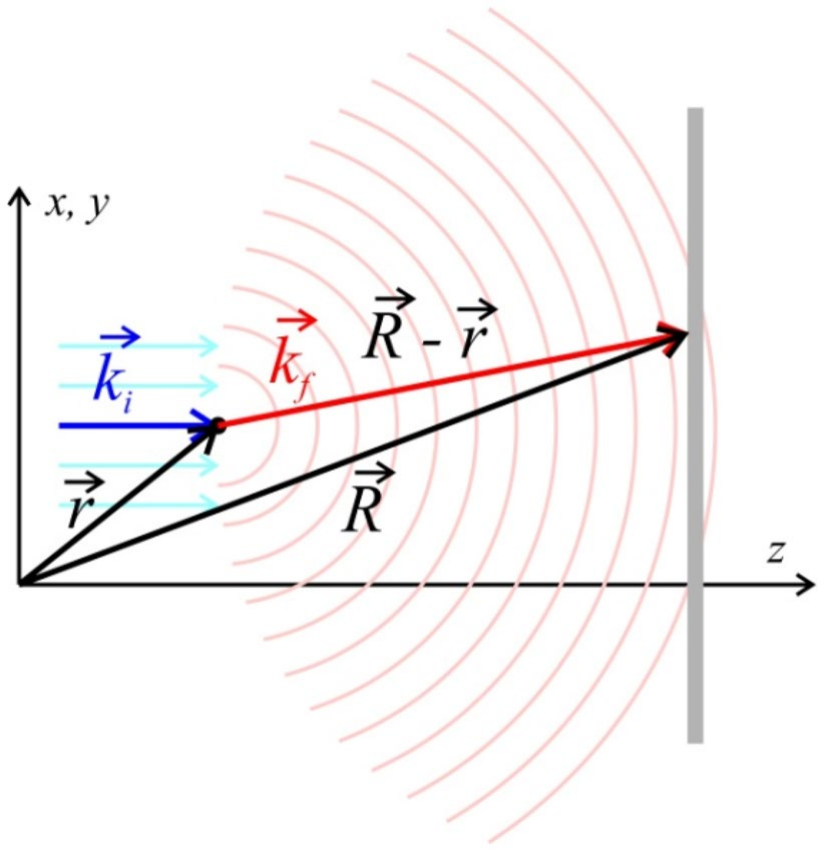
Coordinates and symbols used in calculation of X-ray scattering.

The diffraction pattern of a single lysozyme molecule were calculated by coherently adding waves scattered off individual atoms:

$$I\left( {\vec{R}} \right) = \left| {\sum\limits_{j = 1}^{N} {f_{i,j} \left( q \right)\exp \left( {ikz} \right)\frac{{\exp (ik\left| {\vec{R} - \vec{r}} \right|)}}{{\left| {\vec{R} - \vec{r}} \right|}}} } \right|^{2} ,$$

where $f_{j} \left( q \right)$ is the atomic form factor corresponding to *j*th chemical element:

$$f_{j} \left( q \right) = \sum\limits_{i = 1}^{4} {a_{i} \exp \left[ { - b_{i} \left( {\frac{q}{4\pi }} \right)^{2} } \right]} + c$$

where the parameters $a_{i}$, $b_{i}$, and $c$ are provided in the International Tables for Crystallography^33^; $\vec{r} = \left( {x,y,z} \right)$ is the coordinate of the atom, $\vec{R} = \left( {X,Y,Z} \right)$ is the coordinate on the detector, $\vec{q}$ is the momentum transfer $\vec{q} = \vec{k}_{f} - \vec{k}_{i}$, $\vec{k}_{i} = k\left( {0,0,1} \right)$ is the wave vector of the incident wave and $\vec{k}_{f} = \frac{k}{{\left| {\vec{R} - \vec{r}} \right|}}\left( {X - x,Y - y,Z - z} \right)$ is the wave vector of the scattered wave, $k = \frac{2\pi }{\lambda }$, Fig. 6. The atomic coordinates of the lysozyme molecule were downloaded from the protein database structure 253L^20,21^.

### Electron diffraction pattern of a single virulence protein

The electron diffraction pattern of a single virulence protein was calculated using the following multi-slice simulation protocol: (1) The atomic coordinates of the macromolecule were downloaded from the protein database structure 3J83^23,24^. (2) The sequence of atoms was re-arranged in the order of the increasing $z$-coordinate, and the atoms were numbered as a1, a2 etc. (3) An incident plane wave with a unit amplitude was assumed, $u_{1} (x_{1} ,y_{1} ,z_{1} ) = 1.$ (4) The coordinates of the first atom a1 were read from the text file as $(x_{1}^{(1)} ,y_{1}^{(1)} ,z_{1} )$. (5) The transmission function in the plane at $z_{1}$ was calculated as $t_{1} (x_{1} ,y_{1} ,z_{1} ) = \exp \left[ {i\sigma v_{z} (x_{1} ,y_{1} )} \right]$, where $\sigma$ is the interaction parameter at 200 keV and $v_{z} (x_{1} ,y_{1} )$ is the projected potential of atom a1, calculated from the tabulated parameters corresponding to the chemical elements as described in reference^34^. (6) The exit wave in the plane $(x_{1} ,y_{1} ,z_{1} )$ was calculated as $u^{\prime}_{1} (x_{1} ,y_{1} ,z_{1} ) = u_{1} (x_{1} ,y_{1} ,z_{1} )t_{1} (x_{1} ,y_{1} ,z_{1} ).$(7) The $z$-coordinate of the next atom a2 was read as $z_{2}$, and the distance $\Delta z = z_{2} - z_{1}$ was calculated. (8) The wave function $u^{\prime}_{1} (x_{1} ,y_{1} ,z_{1} )$ was propagated for $\Delta z$ using the angular spectrum method^35^. The resulting wavefront was $u_{2} (x_{2} ,y_{2} ,z_{2} )$. (9) The wave function was propagated through the sample, atom by atom, by repeating steps 4 to 8 until the electron wave had propagated through all the atoms. The obtained distribution is the exit wave. (10) The diffraction pattern was calculated as the square of the amplitude of the Fourier transform of the exit wave.

## Supplementary Information

Supplementary Information.

## Data Availability

All data are available in the main text or the supplementary information.
