# Supplementary material for: Low-dose shift- and rotation-invariant diffraction recognition imaging
Source: Sci Rep. 2022 Jul 1;12:11202. doi: 10.1038/s41598-022-15486-y (PMC9249920; doi:10.1038/s41598-022-15486-y)
Supplement: Supplementary file 1 — Supplementary Information. [file 41598_2022_15486_MOESM1_ESM.docx]

Low-dose shift- and rotation-invariant diffraction recognition imaging

Tatiana Latychevskaia^1,2^* and Alice Kohli^2^

1 Paul Scherrer Institute, Forschungsstrasse 111, 5232 Villigen

2 Physics Department, Winterthurerstrasse 190, 8057 Zurich, University of Zurich

*Corresponding author: Tatiana Latychevskaia, [tatiana.latychevskaia@psi.ch](mailto:tatiana.latychevskaia@psi.ch)

## Diffraction recognition with sorter

Scheme of low-dose diffraction recognition by using a sorting optical element is shown in Fig. S1(a). The sample is a ribosome molecule, structure 6H4N from the protein data bank. The sorter (diffractive optical element) is a binary structure sampled with 1024 × 1024 pixels, and size of 25 × 25 um^2^, thus giving 24.5 nm pixel size, which is a feasible for focussed ion beam nanofabrication (current limit of smallest features is 10 - 15 nm). The distance between the sample and the sorter is 1 mm, and between the sorter and the detector is 0.1 m. When the structure hypothesis is correct, an sharp intensity peak appears in the center of the detector. As the sample rotates the intensity quickly decays, reaching nearly zero within 2° of rotation, Fig. S1(d).


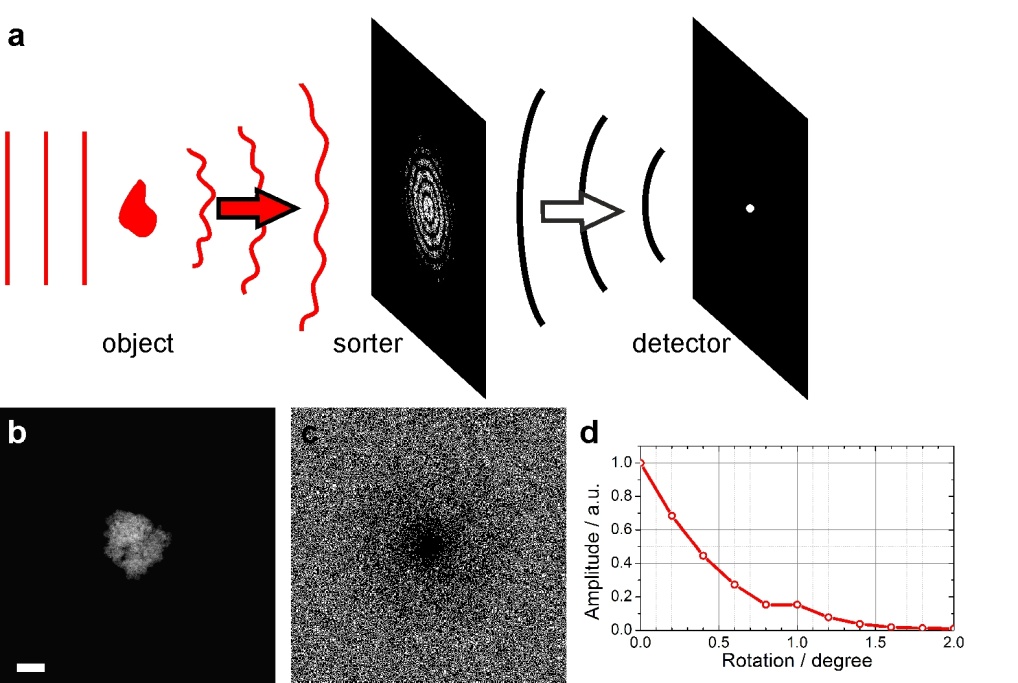


Figure S1. Scheme of low-dose diffraction recognition via using a sorting optical element. (a) Scheme. (b) Phase distribution of the electron exit wave function calculated for 200 keV electrons, ranging from 0 to 1.32 radian; the sample is ribosome macromolecule, the scale bar is 10 nm (c) Sorter, a binary diffractive optical element that focuses the electron beam into convergent wave if the structure hypothesis is correct. (d) Intensity of the peak on the screen as a function of the sample rotation.

## Monte Carlo simulation of 2D image and corresponding 1D radial profiles

Figure Fig. S2 shows Monte Carlo simulation of a 2D distribution of a constant intensity ($I_{0}\left(q,\vartheta\right)=1$) and the corresponding 1D radial profiles calculated as a sum of all counts at a given radial coordinate. It is apparent as the number of counts is increasing, the 2D distribution approaches a uniform constant distribution and the 1D profile approaches the distribution of $I\left(q\right)=\frac{2\pi q}{N}\sum_{n=0}^{N-1} I_{0}\left(q,n\Delta\vartheta\right)=2\pi q$.


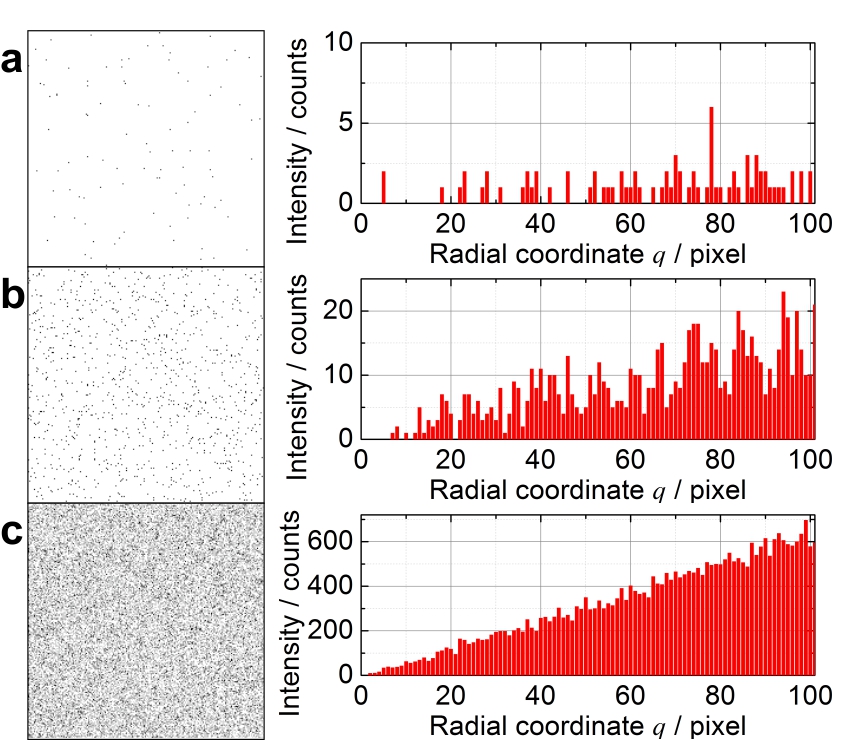


Figure S2. Monte Carlo simulation of a 2D distribution of a constant intensity ($I_{0}\left(q,\vartheta\right)=1$) and the corresponding 1D radial profiles calculated as a sum of all counts at a given radial coordinate. The total number of counts is (a) 1E+2, (b) 1E+3 and (c) 4E+4.

## Effect of the image sizes


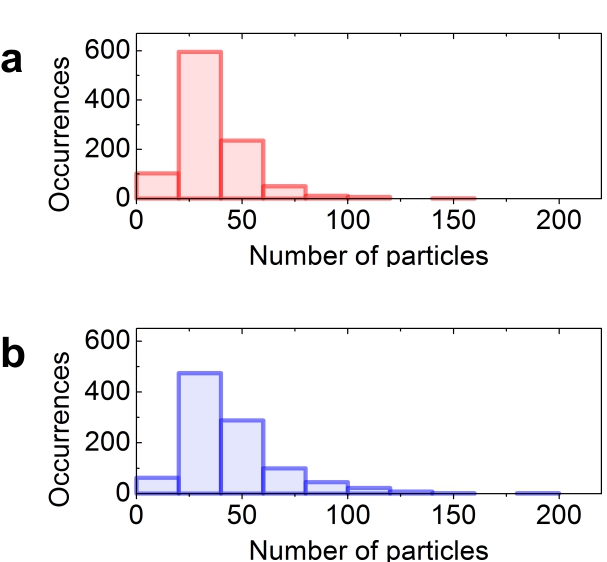


Figure S3. MNIST symbols recognition for different size of the images; the symbols were converted to binary images. The histograms show the number of particles required to achieve 95% probability that the sample structure hypothesis is correct; results of 1000 experiments are shown. The images were sampled with different number of pixels: (a) 28 × 28 pixels, (b) 140 × 140 pixels. Noise was added, SNR = 2. For all three different sizes, about 40 particles were needed to achieve 95% probability that the hypothesis was correct.

## Cross-correlation analysis

For the "cat" cartoon, the recognition analysis was performed using the cross-correlation function (CCF) analysis. For signals $s_{1}\left(x\right)$ and $s_{2}\left(x\right)$, the CCF was calculated as:$\text{CCF}\left(\Delta\right)=\int s_{1}^{*}\left(x\right)s_{2}\left(x+\Delta x\right)\text{d}x$. Here, $s_{1}\left(x\right)$ is the updated radial profile and $s_{2}\left(x\right)$ is the radial profile corresponding to the "up" or "down" conformation. After each detected particle, the updated radial distribution was compared to the "up" and "down" radial distributions by calculating the normalized CCF, , where *n* = 1...*N* and *p* = 1...*N* are the pixel numbers. Thus, two CCF distributions were obtained. Both CCFs exhibit very close values with only about 10E-2 difference, Fig. S4.


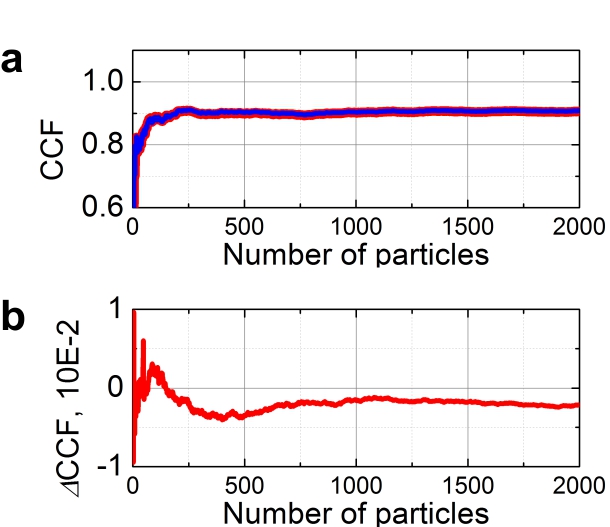


Figure S4. Cross-correlation function (CCF) analysis applied to verify the "up" and "down" hypotheses. (a) CCFs calculated between the one-dimensional radial profile obtained after each detected particle and the one-dimensional radial profiles of the diffraction patterns corresponding to the "up" (red) and "down" (blue) conformations.

(b) Differences between the two CCFs.
